# Associations between Endogenous Dimethylarginines and Renal Function in Healthy Children and Adolescents

**DOI:** 10.3390/ijms131115464

**Published:** 2012-11-21

**Authors:** Aleksandra JaŸwińska-Kozuba, Jens Martens-Lobenhoffer, Andrzej Surdacki, Olga Kruszelnicka, Jarosław Rycaj, Urszula Godula-Stuglik, Stefanie M. Bode-Böger

**Affiliations:** 1 Almed-Elektra Medical Center, 41-710 Ruda Œląska, Poland; E-Mail: almedelektra@interia.pl; 2 Institute for Clinical Pharmacology, Otto-von-Guericke University, 39120 Magdeburg, Germany; E-Mails: jens.martens-lobenhoffer@med.ovgu.de (J.M.-L.); stefanie.bode-boeger@med.ovgu.de (S.M.B.-B.); 3 2nd Department of Cardiology, Jagiellonian University/University Hospital, 31-501 Cracow, Poland; 4 Department of Coronary Artery Disease, the John Paul II Hospital, 31-202 Cracow, Poland; E-Mail: olga.kruszelnicka@gmail.com; 5 Department of Cardiology, Congenital Heart Defects and Electrotherapy, Silesian Center for Heart Diseases in Zabrze, 41-800 Zabrze, Medical University of Silesia, Poland; E-Mail: jacyr@interia.pl; 6 Department of Pediatrics in Zabrze, 41-800 Zabrze, Medical University of Silesia, Poland; E-Mail: kitipnzabrze@sum.edu.pl

**Keywords:** children, asymmetric dimethylarginine, symmetric dimethylarginine, renal function

## Abstract

The structural isomer of asymmetric dimethylarginine (ADMA), symmetric dimethylarginine (SDMA), is eliminated almost entirely by urinary excretion and considered a sensitive index of glomerular filtration rate (GFR). However, reports on this relationship in healthy subjects younger than 18 years of age are rare. Therefore, our aim was to investigate relations between endogenous dimethylarginines and renal function indices in healthy children and adolescents. We studied 40 subjects aged 3–18 years free of coexistent diseases or subclinical carotid atherosclerosis. A serum creatinine-derived estimated GFR (eGFR) was calculated by the revised bedside Schwartz equation. L-arginine, ADMA and SDMA were measured by liquid chromatography-tandem mass spectrometry. Mean eGFR was 122 ± 22 (SD) mL/min per 1.73 m^2^. Creatinine and eGFR exhibited closer correlations with the SDMA/ADMA ratio (*r* = 0.64, *p* < 0.0001; *r* = −0.63, *p* < 0.0001, respectively) than with SDMA (*r* = 0.31, *p* = 0.05; *r* = −0.35, *p* = 0.03). Neither creatinine nor eGFR correlated with ADMA or L-arginine. Adjustment for age or height only slightly attenuated the associations between the SDMA/ADMA ratio and eGFR or creatinine. Our findings suggest the superiority of the SDMA/ADMA ratio over SDMA as a renal function index in healthy children. Thus, further studies are warranted to verify our preliminary results in a larger group of subjects below 18 years of age.

## 1. Introduction

Symmetric dimethylarginine (SDMA), eliminated almost entirely by urinary excretion, is considered an index of glomerular filtration rate (GFR) and is even referred to as an “expensive creatinine” [1]. Irrespective of the still debated pathogenic role of SDMA in patients with renal diseases [2], SDMA appears a more sensitive marker of mild GFR depression than its structural isomer asymmetric dimethylarginine (ADMA), and SDMA levels rise linearly with decreasing GFR at a normal GFR and in mild-to-moderate renal insufficiency [3,4]. According to a meta-analysis [5], SDMA was a good predictor of renal function with an average correlation coefficient (*r*) of 0.77 between a reciprocal of SDMA and various estimates of GFR with even higher *r*-values (0.85) for inulin clearance, the golden standard for measurement of GFR, whereas the respective measures were markedly lower for ADMA.

However, out of 17 full-text source publications underlying this meta-analysis, only one dealt with subjects below 18 years of age [6]. Additionally, that report and the majority of later studies relating renal function to dimethylated L-arginine analogs in children mainly focused on chronic kidney disease (CKD) [6–8] or type 1 diabetes [9–11].

Therefore, our aim was to investigate relations between endogenous dimethylarginines and indices of renal function in healthy children and adolescents.

## 2. Results

Characteristics of the study subjects and plasma levels of L-arginine, ADMA and SDMA are shown in Tables 1 and 2. None of the participants exhibited the presence of carotid plaques or focal intimal thickening.

ADMA and SDMA were mutually interrelated (*r* = 0.61, *p* < 0.0001). SDMA and estimated GFR (eGFR) correlated with homocysteine (*r* = 0.42, *p* = 0.007 and *r* = −0.57, *p* = 0.0001, respectively), and a weak tendency towards a negative relationship between ADMA and age was observed (*r* = −0.22, *p* = 0.17). Averaged intima-media thickness (IMT) of the common carotid artery was unrelated to ADMA, SDMA and eGFR (*p* > 0.25).

**Figure 1:**
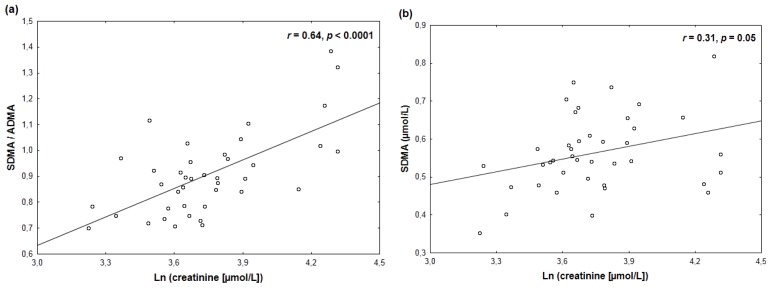
A closer positive correlation of ln-transformed serum creatinine with the SDMA/ADMA ratio (**a**) than with plasma SDMA (**b**).

**Figure 2:**
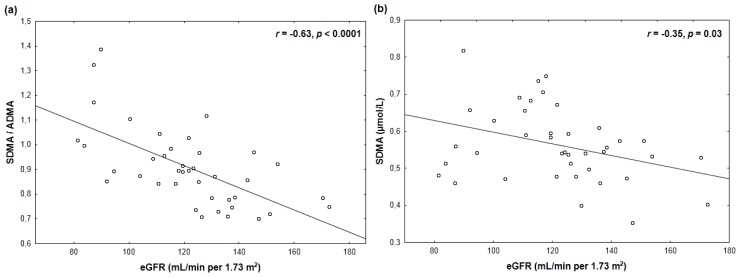
A closer inverse correlation of estimated glomerular filtration rate (eGFR) with the SDMA/ADMA ratio (**a**) than with plasma SDMA (**b**).

Creatinine and eGFR exhibited stronger correlations with the SDMA to ADMA ratio (Table 3; Figure 1a, Figure 2a) than with SDMA (Table 3; Figure 1b, Figure 2b). These differences were even more pronounced after exclusion of two subjects with serum creatinine <26 μmol/L or an eGFR >160 mL/min per 1.73 m^2^ (SDMA/ADMA *vs.* ln (creatinine): *r* = 0.62, *p* < 0.0001; SDMA *vs.* ln (creatinine): *r* = 0.22, *p* = 0.18; SDMA/ADMA *vs.* eGFR: *r* = −0.63, *p* < 0.0001; SDMA *vs.* eGFR: *r* = −0.28, *p* = 0.09). Neither creatinine nor eGFR correlated significantly with ADMA or L-arginine (*p ≥* 0.17) (Table 3).

The associations between the indices of renal function and SDMA or the SDMA/ADMA ratio were only slightly attenuated upon adjustment for age or height (Table 4). The observed relations were not substantially changed on exclusion of two subjects with an elevated level of C-reactive protein (CRP) (>5 mg/L) and after limitation of the analysis either to 33 boys or to 30 subjects with a negative parental history of premature coronary artery disease.

## 3. Discussion

Our salient observation was a closer association of renal function indices with the SDMA/ADMA ratio than with SDMA in children and adolescents free of coexistent diseases or subclinical carotid atherosclerosis.

### 3.1. Comparison with Other Studies Relating Renal Function to Endogenous Dimethylarginines in Children

Plasma L-arginine, ADMA and SDMA levels in the study participants were similar to those previously reported for liquid or gas chromatography–tandem mass spectrometry in healthy children [7,12–14]. A positive correlation between SDMA and homocysteine might have been due to the dependence of both these parameters on renal function.

Our findings are partially consistent with the report by Brooks *et al.*[7] who observed that creatinine-derived eGFR correlated with SDMA (*r* = −0.73), SDMA/ADMA ratio (*r* = −0.74) and only weakly with ADMA (*r* ≈ −0.33) in data pooled from 28 children and adolescents aged 13 ± 1 years with stage 2–3 CKD and 10 healthy age-matched siblings. Intriguingly, 15 years ago an early paper by Goonasekera *et al.*[6] described a closer correlation of ADMA (*r* = −0.77) than SDMA (*r* = −0.38) with eGFR derived from plasma creatinine by the Morris formula in 38 hypertensive children and adolescents aged 1–18 years (median, 8 years) with mainly nephrogenic hypertension and mildly depressed eGFR.

We observed a weaker correlation between eGFR and SDMA (*r* = −0.35) than estimated according to a meta-analysis by Kielstein *et al.*[5] on the basis of 18 studies involving a total of 2136 subjects. Nevertheless, that meta-analysis focused mainly on adult subjects and our results are in keeping with data by Marcovecchio *et al.*[10] who reported a similar *r-*value (−0.38) between SDMA and plasma clearance of Inutest—branched chain polyfructosan with a Stokes radius profile equivalent to inulin—in 183 children aged 14.8 ± 4.1 years with type 1 diabetes and a mean eGFR of 149 ± 32 mL/min per 1.73 m^2^ of body-surface area. Thus, the present study has extended the evidence supporting potential utility and limitations of an assay of endogenous dimethylarginines as an index of eGFR to healthy children. Nevertheless, our preliminary data require validation in a larger group of subjects.

### 3.2. Proposed Mechanisms of the Close Relationship between Renal Function Indices and the SDMA to ADMA Ratio

A close inverse correlation between eGFR and the SDMA/ADMA ratio might appear unexpected because an elevated ADMA/SDMA ratio had been previously suggested as a hallmark of depressed activity of cytosolic dimethylarginine dimethylaminohydrolases (DDAHs) [1,15], a family of cytosolic enzymes which hydrolyze over 80% of ADMA generated daily to L-citrulline and dimethylamine but are inactive towards SDMA [16–20]. However, this concept emerged from early reports on elevated ADMA in subjects with a given disease or risk factor with reference to matched healthy controls [15,21,22] usually exhibiting unchanged eGFR, the major determinant of SDMA levels. This preferential ADMA accumulation was putatively linked to depressed DDAH activity in experimental models of the disease [18–20]. Nevertheless, our study subjects exhibited no evidence of abnormalities in which excessive ADMA accumulation or DDAH down-regulation had previously been shown [19,20,23]. Accordingly, it might be hypothesized that the effect of renal function on the inter-individual variability of the SDMA/ADMA ratio might have been more pronounced in this selected group of subjects. In addition, the calculation of the ratio could better reflect renal function because this approach probably accentuated the contribution of the renal ability to excrete SDMA (closely linked to GFR values) upon adjustment to the levels of ADMA, the vast majority of which is metabolized by DDAHs [17], *i.e.*, independently of GFR.

In the present study, eGFR and ADMA were unrelated, whereas SDMA and ADMA were positively and moderately correlated with each other. As pointed out by Kielstein *et al.*[5], both ADMA and SDMA are produced in every nucleated cell, being liberated during catabolism of proteins with dimethylated arginine residues. Although there are different profiles of methyl-accepting protein substrates for protein arginine methyltransferases type I and type II (catalyzing ADMA and SDMA formation, respectively) [24], it has long been recognized that cultured endothelial cells release both ADMA and—albeit less—also SDMA [25,26]. Therefore, generation of both free ADMA and SDMA is dependent on the activity of protein arginine methyltransferases and related to protein turnover rate [27], whereas SDMA concentrations are also influenced by renal function, which provides a rationale for the ability of the SDMA/ADMA ratio to distinguish a relatively “pure” effect of GFR.

### 3.3. Study Limitations

That we have based eGFR exclusively on serum creatinine, constitutes a major limitation of the present study, in addition to a small number of the study participants. Tutarel *et al.*[28] demonstrated that SDMA exhibited a markedly better correlation with cystatin C-based eGFR (by the Larsson formula) than eGFR calculated from serum creatinine by the Chronic Kidney Disease Epidemiology Collaboration (CKD-EPI) or the Modification of Diet in Renal Disease (MDRD) study equations in 97 young adults with congenital heart disease and a normal eGFR in almost all of the subjects. On the other hand, Marcovecchio *et al.*[10] reported a similar magnitude of moderate correlations of Inutest-derived GFR with SDMA and creatinine-derived eGFR in children with type 1 diabetes and normal or elevated eGFR due to hyperfiltration. A recent paper by Wasilewska *et al.*[8] suggested even better performance of SDMA than cystatin C for the detection of early CKD stages in children. However, in that report ADMA was not measured and SDMA was quantified by an enzyme-linked immunosorbent assay [8], considered less reliable than liquid chromatography-tandem mass spectrometry for detection of intergroup differences in dimethylarginines [29–31]. Therefore, future comparisons of SDMA and the SDMA/ADMA ratio with the reference to GFR measures based also on other markers than serum creatinine might provide further insight into a potential utility of these indices for renal function assessment in children and adolescents.

Although exclusively healthy children were entered into the study, it cannot be excluded that some of them might have exhibited accelerated early atherogenesis, known to be associated with increased ADMA levels not only in adults [32] but also in pre-pubertal children [33], which could be a potential additional source of data variability. However, the absence of subclinical carotid atherosclerosis was confirmed by ultrasound and averaged common carotid IMT remained within the previously reported normal range [34] in all the study participants.

## 4. Experimental Section

### 4.1. Study Subjects

We studied 40 healthy children and adolescents (33 boys and seven girls) aged 3.4–17.9 years (median, 10.6 years). Exclusion criteria included congenital heart or pulmonary defects, clinical or biochemical evidence of renal or hepatic pathology, hypertension, diabetes, obesity, and any other significant chronic coexistent diseases, acute disorders or relevant abnormalities in routine blood or urine analyses. Additionally, those with ultrasound evidence of atherosclerotic plaques in carotid arteries were also excluded.

### 4.2. Study Protocol

The study was performed in accordance with the Helsinki Declaration, the protocol had been approved by the ethics committee of the Medical University of Silesia and written informed consent was obtained from the parents of each participant.

Demographical data and medical history were recorded according to a pre-specified questionnaire and anthropometric measurements (height, weight, waist circumference) were performed. Additionally, ultrasonography of carotid arteries was performed to confirm the absence of atherosclerotic plaques.

On a separate day in the morning, about 10 mL of blood was drawn from an antecubital vein after overnight fast into sampling tubes containing ethylenediaminetetraacetic acid or no anticoagulant, centrifuged and the collected portions of serum and plasma were frozen initially at −20 °C (and −70 °C in case of prolonged storage of samples) until assayed.

Biochemical analyses included lipids, creatinine, glucose, homocysteine and CRP. Creatinine was measured by the Jaffe assay with the isotope dilution mass spectrometry (IDMS)-traceable calibration (Roche Hitachi Chemistry Analyzer, Roche Diagnostics, Basel, Switzerland). This method exhibited a percent bias (with 95% confidence intervals) of no more than 5% with the reference to IDMS for serum creatinine below 150 μmol/L [35]. CRP and homocysteine were quantified by immunoturbidometry (Roche Diagnostics) and a chemiluminescent microparticle immunoassay (Abbott Diagnostics, Abbott Park, IL, USA), respectively.

Plasma concentrations of L-arginine, ADMA and SDMA were measured by means of liquid chromatography-electrospray tandem mass spectrometry methods with an isotope-labeled internal standard as described in detail elsewhere [36]. The imprecision of this method was 4.5%, 5.5% and 3.9% for L-arginine, ADMA and SDMA, respectively, with accuracies better than 5% for all the substances.

An eGFR was calculated from serum creatinine and the height by the revised bedside Schwartz equation [37], which has been validated also for children and adolescents with normal renal function [38].

The common carotid artery, carotid bulb and internal carotid artery were visualized on both sides by B-mode imaging in the longitudinal plane using a high-resolution ultrasound device (iU22 xMATRIX Ultrasound System, Philips Healthcare, Best, The Netherlands) with a 12-MHz linear digital ultrasound probe by an investigator (Jarosław Rycaj) who was unaware of characteristics of the subjects. The image was recorded and stored for off-line analysis by manual tracing to measure IMT. As proposed previously [39], plaques were defined as focal structures encroaching into the arterial lumen of at least 0.5 mm or 50% of the surrounding IMT value. IMT was measured at end-diastole on the far wall of the common carotid artery within a 1 cm segment immediately proximal to the carotid bulb and the final value was averaged from three measurements per each side [39].

### 4.3. Statistical Analysis

Values are expressed as means ± SD (standard deviation) for continuous variables with normal distribution, medians (interquartile range) for not normally distributed continuous data, and numbers (%) for categorical variables. The accordance with a normal distribution was checked by the Lilliefors test. Bivariate correlations were estimated by Pearson’s correlation coefficients (*r*). In order to obtain a normal distribution, logarithmic transformation (ln, natural logarithm) was applied when necessary. To test an independent relationship between selected parameters, multiple regression was applied and mean standardized regression coefficients (β), their standard errors (SEM) and respective *p*-values for individual variables were shown. A *p*-value below 0.05 was inferred significant. All statistical tests were performed using STATISTICA (data analysis software system, version 10.0; StatSoft, Inc., Tulsa, Oklahoma, USA, 2011).

## 5. Conclusions

Our findings suggest the superiority of the SDMA/ADMA ratio over SDMA as an index of renal function in healthy children and adolescents. Nevertheless, further studies are warranted to verify these preliminary results in a large cohort of subjects below 18 years of age.

## Figures and Tables

**Table 1:** Clinical and biochemical characteristics of 40 study subjects.

Characteristic	
Age (years)	10.1 ± 3.6
Male gender (M/F)	33/7
Parental history of premature coronary artery disease	10 (25%)
Height (percentiles)	48 ± 28
Weight (percentiles)	43 ± 24
Waist circumference (cm)	63 (56–74)
Creatinine (μmol/L)	40.2 (36.2–49.0)
Estimated glomerular filtration rate (mL/min per 1.73 m^2^)	122.4 (109.7–136.1)
Low-density lipoproteins-cholesterol (mmol/L)	2.2 (1.8–2.7)
High-density lipoproteins-cholesterol (mmol/L)	1.5 (1.3–1.8)
Triglycerides (mmol/L)	0.72 (0.54–0.94)
Glucose (mmol/L)	4.6 (4.4–5.1)
Homocysteine (μmol/L)	8.5 (7.4–10.1)
Averaged intima-media thickness of the common carotid artery (mm)	0.45 (0.41–0.53)

Data are shown as means ± SD, medians (interquartile range) or *n* (%).

**Table 2:** Plasma levels of L-arginine, asymmetric dimethylarginine (ADMA) and symmetric dimethylarginine (SDMA).

Metabolite	Mean ± SD
l-arginine (μmol/L)	69 ± 22
ADMA (μmol/L)	0.63 ± 0.12
SDMA (μmol/L)	0.56 ± 0.10
SDMA/ADMA ratio	0.91 ± 0.16

ADMA: asymmetric dimethylarginine; SDMA: symmetric dimethylarginine.

**Table 3:** Pearson’s correlation coefficients between renal indices and ADMA, SDMA and L-arginine.

	eGFR	Ln (creatinine)
ADMA	0.19	−0.22
SDMA	−0.35 **	0.31 *
SDMA/ADMA ratio	−0.63 ***	0.64 ***
l-arginine	0.02	−0.01

* *p* = 0.05,

** *p* = 0.03,

*** *p* < 0.0001.

ADMA: asymmetric dimethylarginine; eGFR: estimated glomerular filtration rate; SDMA: symmetric dimethylarginine.

**Table 4:** Effects of adjustment for age and height on the relations between indices of renal function and endogenous dimethylarginines.

Relationship	Mean standardized regression coefficient (β) ± SEM (*p*-values in parentheses)		
	
	Unadjusted	Age-adjusted	Height-adjusted
SDMA *vs.* eGFR	−0.35 ± 0.15 (0.03)	−0.36 ± 0.18 (0.06)	−0.33 ± 0.18 (0.08)
SDMA/ADMA *vs.* eGFR	−0.63 ± 0.13 (<0.0001)	−0.52 ± 0.15 (0.001)	−0.52 ± 0.14 (0.0008)
SDMA *vs.* ln (creatinine)	0.31 ± 0.15 (0.05)	0.52 ± 0.27 (0.06)	0.45 ± 0.28 (0.12)
SDMA/ADMA *vs.* ln (creat.)	0.64 ± 0.12 (<0.0001)	0.75 ± 0.22 (0.001)	0.80 ± 0.22 (0.0009)

ADMA: asymmetric dimethylarginine; creat.: creatinine; eGFR: estimated glomerular filtration rate; SDMA: symmetric dimethylarginine; SEM: standard error of the mean.

## References

[1] Bode-Böger S.M., Scalera F., Kielstein J.T., Martens-Lobenhoffer J., Breithardt G., Fobker M., Reinecke H. (2006). Symmetrical dimethylarginine: A new combined parameter for renal function and extent of coronary artery disease. J. Am. Soc. Nephrol.

[2] Kielstein J.T., Fliser D., Veldink H. (2009). Asymmetric dimethylarginine and symmetric dimethylarginine: axis of evil or useful alliance?. Semin. Dial.

[3] Marescau B., Nagels G., Possemiers I., De Broe M.E., Becaus I., Billiouw J.M., Lornoy W., De Deyn P.P. (1997). Guanidino compounds in serum and urine of nondialyzed patients with chronic renal insufficiency. Metabolism.

[4] Fliser D., Kronenberg F., Kielstein J.T., Morath C., Bode-Böger S.M., Haller H., Ritz E. (2005). Asymmetric dimethylarginine and progression of chronic kidney disease: The mild to moderate kidney disease study. J. Am. Soc. Nephrol.

[5] Kielstein J.T., Salpeter S.R., Bode-Böger S.M., Cooke J.P., Fliser D. (2006). Symmetric dimethylarginine (SDMA) as endogenous marker of renal function—A meta-analysis. Nephrol. Dial. Transplant.

[6] Goonasekera C.D., Rees D.D., Woolard P., Frend A., Shah V., Dillon M.J. (1997). Nitric oxide synthase inhibitors and hypertension in children and adolescents. J. Hypertens.

[7] Brooks E.R., Langman C.B., Wang S., Price H.E., Hodges A.L., Darling L., Yang A.Z., Smith F.A. (2009). Methylated arginine derivatives in children and adolescents with chronic kidney disease. Pediatr. Nephrol.

[8] Wasilewska A., Taranta-Janusz K., Zoch-Zwierz W., Michaluk-Skutnik J. (2012). Is plasma symmetric dimethylarginine a suitable marker of renal function in children and adolescents?. Scand. J. Urol. Nephrol.

[9] Heilman K., Zilmer M., Zilmer K., Kool P., Tillmann V. (2009). Elevated plasma adiponectin and decreased plasma homocysteine and asymmetric dimethylarginine in children with type 1 diabetes. Scand. J. Clin. Lab. Invest.

[10] Marcovecchio M.L., Dalton R.N., Turner C., Prevost A.T., Widmer B., Amin R., Dunger D.B. (2010). Symmetric dimethylarginine, an endogenous marker of glomerular filtration rate, and the risk for microalbuminuria in young people with type 1 diabetes. Arch. Dis. Child.

[11] Marcovecchio M.L., Widmer B., Turner C., Dunger D.B., Dalton R.N. (2011). Asymmetric dimethylarginine in young people with Type 1 diabetes: A paradoxical association with HbA(1c). Diabet. Med.

[12] Lücke T., Kanzelmeyer N., Kemper M.J., Tsikas D., Das A.M. (2007). Developmental changes in the L-arginine/nitric oxide pathway from infancy to adulthood: Plasma asymmetric dimethylarginine levels decrease with age. Clin. Chem. Lab. Med.

[13] Huemer M., Simma B., Mayr D., Mühl A., Rami B., Schober E., Ulmer H., Zanier U., Bodamer O.A. (2011). Low levels of asymmetric dimethylarginine in children with diabetes mellitus type I compared with healthy children. J. Pediatr.

[14] Aldámiz-Echevarría L., Andrade F. (2012). Asymmetric dimethylarginine, endothelial dysfunction and renal disease. Int. J. Mol. Sci.

[15] Abbasi F., Asagmi T., Cooke J.P., Lamendola C., McLaughlin T., Reaven G.M., Stühlinger M., Tsao P.S. (2001). Plasma concentrations of asymmetric dimethylarginine are increased in patients with type 2 diabetes mellitus. Am. J. Cardiol.

[16] Ogawa T., Kimoto M., Sasaoka K. (1989). Purification and properties of a new enzyme, *N**^G^**,N**^G^*-dimethylarginine dimethylaminohydrolase, from rat kidney. J. Biol. Chem.

[17] Achan V., Broadhead M., Malaki M., Whitley G., Leiper J., MacAllister R., Vallance P. (2003). Asymmetric dimethylarginine causes hypertension and cardiac dysfunction in humans and is actively metabolized by dimethylarginine dimethylaminohydrolase. Arterioscler. Thromb. Vasc. Biol.

[18] Vallance P., Leiper J. (2004). Cardiovascular biology of the asymmetric dimethylarginine: Dimethylarginine dimethylaminohydrolase pathway. Arterioscler. Thromb. Vasc. Biol.

[19] Palm F., Onozato M.L., Luo Z., Wilcox C.S. (2007). Dimethylarginine dimethylaminohydrolase (DDAH): Expression, regulation, and function in the cardiovascular and renal systems. Am. J. Physiol. Heart Circ. Physiol.

[20] Teerlink T., Luo Z., Palm F., Wilcox C.S. (2009). Cellular ADMA: Regulation and action. Pharmacol. Res.

[21] Böger R.H., Bode-Böger S.M., Szuba A., Tsao P.S., Chan J.R., Tangphao O., Blaschke T.F., Cooke J.P. (1998). Asymmetric dimethylarginine (ADMA): A novel risk factor for endothelial dysfunction: Its role in hypercholesterolemia. Circulation.

[22] Surdacki A., Nowicki M., Sandmann J., Tsikas D., Böger R.H., Bode-Böger S.M., Kruszelnicka-Kwiatkowska O., Kokot F., Dubiel J.S., Frölich J.C. (1999). Reduced urinary excretion of nitric oxide metabolites and increased plasma levels of asymmetric dimethylarginine in men with essential hypertension. J. Cardiovasc. Pharmacol.

[23] Surdacki A. (2008). L-arginine analogs—Inactive markers or active agents in atherogenesis?. Cardiovasc. Hematol. Agents Med. Chem.

[24] Gary J.D., Clarke S. (1998). RNA and protein interactions modulated by protein arginine methylation. Prog. Nucleic. Acid Res. Mol. Biol.

[25] Fickling S.A., Leone A.M., Nussey S.S., Vallance P., Whitley G.St.J. (1993). Synthesis of *N^G^, N^G^* dimethylarginine by human endothelial cells. Endothelium.

[26] Böger R.H., Bode-Böger S.M., Tsao P.S., Lin P.S., Chan J.R., Cooke J.P. (2000). An endogenous inhibitor of nitric oxide synthase regulates endothelial adhesiveness for monocytes. J. Am. Coll. Cardiol.

[27] Marliss E.B., Chevalier S., Gougeon R., Morais J.A., Lamarche M., Adegoke O.A., Wu G. (2006). Elevations of plasma methylarginines in obesity and aging are related to insulin sensitivity and rates of protein turnover. Diabetologia.

[28] Tutarel O., Denecke A., Bode-Böger S.M., Martens-Lobenhoffer J., Schieffer B., Westhoff-Bleck M., Kielstein J.T. (2011). Symmetrical dimethylarginine outperforms CKD-EPI and MDRD-derived eGFR for the assessment of renal function in patients with adult congenital heart disease. Kidney Blood Press Res.

[29] Martens-Lobenhoffer J., Westphal S., Awiszus F., Bode-Böger S.M., Luley C. (2005). Determination of asymmetric dimethylarginine: Liquid chromatography-mass spectrometry or ELISA?. Clin. Chem.

[30] Valtonen P., Karppi J., Nyyssonen K., Valkonen V.P., Halonen T., Punnonen K. (2005). Comparison of HPLC method and commercial ELISA assay for asymmetric dimethylarginine (ADMA) determination in human serum. J. Chromatogr. B.

[31] Horowitz J.D., Heresztyn T. (2007). An overview of plasma concentrations of asymmetric dimethylarginine (ADMA) in health and disease and in clinical studies: Methodological considerations. J. Chromatogr. B.

[32] Miyazaki H., Matsuoka H., Cooke J.P., Usui M., Ueda S., Okuda S., Imaizumi T. (1999). Endogenous nitric oxide synthase inhibitor: A novel marker of atherosclerosis. Circulation.

[33] Ayer J.G., Harmer J.A., Nakhla S., Xuan W., Ng M.K., Raitakari O.T., Marks G.B., Celermajer D.S. (2009). HDL-cholesterol, blood pressure, and asymmetric dimethylarginine are significantly associated with arterial wall thickness in children. Arterioscler. Thromb. Vasc. Biol.

[34] Urbina E.M., Williams R.V., Alpert B.S., Collins R.T., Daniels S.R., Hayman L., Jacobson M., Mahoney L., Mietus-Snyder M., Rocchini A. (2009). Noninvasive assessment of subclinical atherosclerosis in children and adolescents: Recommendations for standard assessment for clinical research: A scientific statement from the American Heart Association. Hypertension.

[35] Peake M., Whiting M. (2006). Measurement of serum creatinine—Current status and future goals. Clin. Biochem. Rev.

[36] Martens-Lobenhoffer J., Bode-Böger S.M. (2006). Fast and efficient determination of arginine, symmetric dimethylarginine, and asymmetric dimethylarginine in biological fluids by hydrophilic-interaction liquid chromatography-electrospray tandem mass spectrometry. Clin. Chem.

[37] Schwartz G.J., Muñoz A., Schneider M.F., Mak R.H., Kaskel F., Warady B.A., Furth S.L. (2009). New equations to estimate GFR in children with CKD. J. Am. Soc. Nephrol.

[38] Staples A., LeBlond R., Watkins S., Wong C., Brandt J. (2010). Validation of the revised Schwartz estimating equation in a predominantly non-CKD population. Pediatr. Nephrol.

[39] Touboul P.-J., Hennerici M.G., Meairs S., Adams H., Amarenco P., Bornstein N., Csiba L., Desvarieux M., Ebrahim S., Fatar M. (2007). Mannheim carotid intima-media thickness consensus (2004–2006). Cerebrovasc. Dis.

